# ECMO-Therapie bei COVID-19-ARDS in der Schwangerschaft ermöglicht den Erhalt einer Schwangerschaft mit termingerechter Entbindung

**DOI:** 10.1007/s00101-022-01232-6

**Published:** 2022-12-08

**Authors:** Magdalena Sitter, Corinna Fröhlich, Peter Kranke, Christian Markus, Achim Wöckel, Monika Rehn, Catharina Bartmann, Eric Frieauff, Patrick Meybohm, Ulrich Pecks, Daniel Röder

**Affiliations:** 1 Klinik und Poliklinik für Anästhesiologie, Intensivmedizin, Notfallmedizin und Schmerztherapie, Universitätsklinikum Würzburg, Oberdürrbacher Str. 6, 97080 Würzburg, Deutschland; 2 Frauenklinik und Poliklinik, Universitätsklinikum Würzburg, Würzburg, Deutschland; 3 Kinderklinik und Poliklinik, Universitätsklinikum Würzburg, Würzburg, Deutschland; 4 Klinik für Gynäkologie und Geburtshilfe, Universitätsklinikum Schleswig-Holstein, Campus Kiel, Kiel, Deutschland

## Falldarstellung

### Anamnese

Die 24-jährige Erstgravida stellte sich in der 26. Schwangerschaftswoche (SSW) (25 + 3 SSW) in einem externen Krankenhaus der Schwerpunktversorgung vor. Seit 5 Tagen bestünden ein bellender, trockener Husten, Halsschmerzen, Schmerzen im unteren Rücken, Fieber und Schüttelfrost. Ein ambulant durchgeführter PCR-Test auf SARS-CoV‑2 war bereits positiv ausgefallen. Der bisherige Schwangerschaftsverlauf war unauffällig. An Vorerkrankungen lag eine Adipositas per magna (BMI 46,6 kg/m^2^) vor.

Die Patientin präsentierte sich bei der stationären Aufnahme tachykard (Herzfrequenz (HF) 147/min) und tachypnoisch mit zunehmender Dyspnoe. Die Diagnose SARS-CoV-2-Pneumonie wurde klinisch, d. h. unter Verzicht auf ionisierende Strahlung, gestellt. Bei respiratorischer Partialinsuffizienz erhielt die Patientin 3 l/min Sauerstoffinsufflation via Nasenbrille. Zudem wurden eine Prednisolontherapie (40 mg/Tag) und eine therapeutische Antikoagulation mit niedermolekularem Heparin begonnen. Die respiratorische Situation der schwangeren Patientin verschlechterte sich im Verlauf (Atemfrequenz (AF) 30/min, Sauerstoffsättigung (S_p_O_2_) 92 % unter 3 l/min O_2_-Insufflation), so dass wir sie auf Bitte der Quellklinik am 3. Tag nach Hospitalisierung und 8. Tag nach Symptombeginn in unser ECMO(extrakorporale Membranoxygenierung)-Zentrum übernahmen.

### Klinischer Befund

Bei Aufnahme auf die Intensivstation (ICU Tag 1) war die Patientin leicht agitiert (RASS (Richmond Agitation Sedation Scale) +1) und unter Spontanatmung und Sauerstoffinsufflation via Nasenbrille von 8 l/min tachypnoisch (AF 40/min). In der Sonographie präsentierte sich der Fetus zeitgerecht entwickelt und mit guten Kindsbewegungen.

### Diagnose

**Figure 1:**
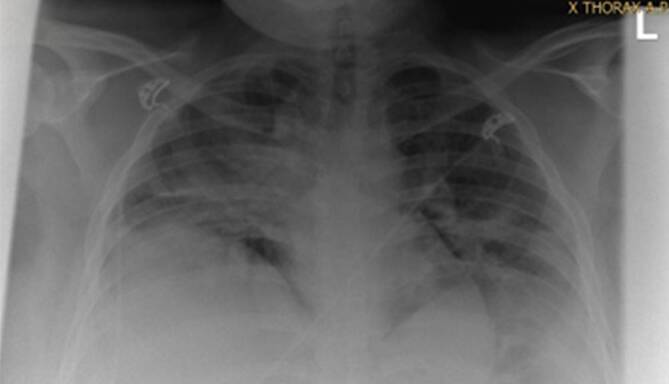

In der Röntgenübersichtsaufnahme des Thorax zeigte sich eine streifige Transparenzminderung (Abb. 1). Die arteriellen Blutgasanalysen (BGA) wiesen eine respiratorische Partialinsuffizienz (p_a_O_2_ 60,7 mm Hg, p_a_CO_2_ 24,5 mm Hg), bei noch kompensierten pH- (7,40) und Lactatwerten (1,7 mmol/l) aus. Die Infektionsparameter waren moderat erhöht (Leukozyten 11/nl (5–10/nl), CRP 6,48 mg/dl (0–0,5 mg/dl), Interleukin‑6 25,8 pg/ml (0–7 pg/ml), Ferritin 305 µg/l (15–150 µg/l)). Das Prokalzitonin war im Einklang mit einer Virusinfektion normwertig (0,39 ng/ml (0–0,5 ng/ml)), sodass in der Zusammenschau der Befunde die Diagnose SARS-CoV-2-Pneumonie bestätigt werden konnte.

### Therapie und Verlauf

Die fetalen Herztöne und die maternale Wehentätigkeit wurden täglich via Kardiotokographie (CTG) überwacht und waren stets unauffällig. Regelmäßige sonographische Biometrien zeigten einen zeitgerecht entwickelten und vitalen Fetus. Die extern begonnene Kortikoidtherapie mit Prednisolon wurde weitergeführt. Im interdisziplinären Konsens (Anästhesiologie, Geburtshilfe, Neonatologie) wurde im Rahmen einer Risiko-Nutzen-Abwägung zunächst auf eine operative Entbindung via Sectio caesarea verzichtet. Als Zielkriterien wurden arterielle pO_2_-Werte von mindestens 70 mm Hg und ein Hämoglobinwert von mindestens 8,5 g/dl angestrebt. Des Weiteren wurde leitliniengemäß die fetale Lungenreife mittels 2‑maliger Gabe von 12 mg Betamethason i.m. im Abstand von 24 h induziert [1].

Trotz Sauerstoffinsufflation via Nasenbrille und – soweit toleriert – intermittierend via Maske (bis zu 15 l/min) nahm am ICU-Tag 2 die respiratorische Insuffizienz zu (pO_2_ 59,3 mm Hg, pCO_2_ 23,8 mm Hg, Lactat 0,9 mmol/l, AF 52/min), sodass zunächst eine High-Flow-Sauerstofftherapie (F_I_O_2_ 100 %, 45 l/min) begonnen und intermittierend eine nichtinvasive Maskenbeatmung durchgeführt wurde (F_I_O_2_ 100 %, p_mean_ 3–7 mbar, CPAP 3–5 mbar).

Aufgrund einer zunehmenden Hypoxämie (pO_2_ 50,6 mm Hg, pCO_2_ 34,9 mm Hg, Lactat 0,8 mmol/l) unter muskulärer Erschöpfung und Ablehnung der nichtinvasiven Maskenbeatmung an ICU-Tag 4 wurde entschieden, die wache, spontan atmende Patientin an eine venovenöse (vv‑)ECMO anzuschließen. Während der Kanülierung kam es zu einer weiteren Aggravation der Hypoxämie, die die unmittelbare Intubation erforderte. Nach Narkoseeinleitung ohne Katecholaminbedarf wurde die Patientin komplikationslos intubiert und druckkontrolliert beatmet (PCV, F_I_O_2_ 100 %, PEEP 15 mbar, p_peak_ 35 mbar). Nach Intubation wurde die ECMO-Anlage komplettiert. Unter der ECMO-Therapie erfolgte für 3 Tage eine jeweils 18 h andauernde, wechselseitige 135°-Lagerung. Die Patientin wurde tief sediert (Ziel-RASS −5) und weiterhin unter Beachtung lungenprotektiver Druckniveaus kontrolliert bzw. unterstützt beatmet (F_I_O_2_ 50 %, PEEP 15 mbar, p_max_ 30 mbar).

Die Oxygenierung besserte sich rasch unter ECMO-, Beatmungs- und Lagerungstherapie, sodass die Blut- und „Sweep-gas“-Flüsse der ECMO schrittweise reduziert und die ECMO-Kanülen nach 5 Tagen (ICU-Tag 8) entfernt wurden (Tab. 1). Die BGA unmittelbar vor Beendigung der ECMO-Therapie zeigte unter druckunterstützter Spontanatmung einen kompensierten Gasaustausch und einen ausgeglichenen Säure-Basen-Haushalt (pO_2_ 83,4 mm Hg, pCO_2_ 44,2 mm Hg, pH 7,44, Lactat 0,9 mmol/l).

**Table 1:** 

ECMO-Therapie 5 Tage, 101,5 h	Tag 1	Tag 2	Tag 3	Tag 4	Tag 5
ICU-Tag	4	5	6	7	8
Beatmungsmodus	PCV	PCV	PCV/PSV	PSV	PSV
p_insp_ (mbar)	23	23	18–23	17–22	18–22
PEEP (mbar)	15	15	15	14	15
F_I_O_2_ (%)	50	50–60	50	40–50	40
p_a_O_2_-Range (mm Hg) Median 24 h (mm Hg)	43,6–112 80,3	67,3–138 93,2	72,1–107 93,8	79,7–98,4 88,3	64,5–107 82,4
p_a_O_2_/F_I_O_2_− IQR (mm Hg) Median 24 h (mm Hg)	106–201 168,9	118–205 151	102–223 163,4	140–205 176,5	157–228 180,1
Kanülierung	V. jugularis rechts V. femoralis rechts	–	–	–	Entfernung
Blutfluss (l/min)	3,2	2,9–3,3	3,1–3,4	2,0–3,1	1,9
„Sweep-gas“-Fluss (l/min)	1–2	2–4	3	2	1–2

*ECMO* Extrakorporale Membranoxygenierung, *ICU* Intensive Care Unit, *p*_*insp*_ inspiratorischer Beatmungsdruck, *PEEP* positiver endexspiratorischer Druck, *F*_*I*_*O*_*2*_ inspiratorische Sauerstofffraktion, *p*_*a*_*O*_2_ arterieller Sauerstoffpartialdruck, *IQR* Interquartilsabstand, *PCV* „pressure controlled ventilation“ (druckkontrollierte Beatmung), *PSV* „pressure support ventilation“ (druckunterstützte Beatmung)

**Figure 2:**
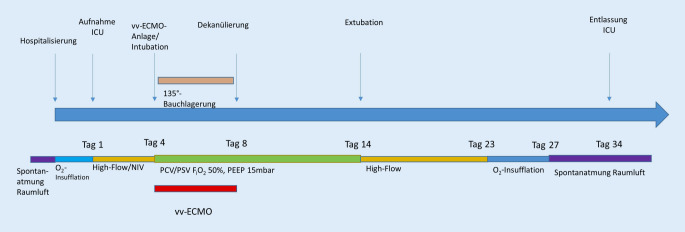

Nach weiteren 6 Tagen druckunterstützter Beatmung wurde die Patientin schrittweise vom Ventilator entwöhnt und am ICU-Tag 14 erfolgreich extubiert. Am ICU-Tag 35 konnte sie spontan atmend, mit unauffälligen Atemgeräuschen und einer S_p_O_2_ von 98 % unter Raumluft auf die geburtshilfliche Normalstation verlegt werden. Der zeitliche Verlauf der ARDS(Acute Respiratory Distress Syndrome)-Behandlung ist in Abb. 2 dargestellt.

Der Kreislauf wurde lediglich tageweise mit niedrigen Dosen Noradrenalin (bis max. 7 µg/min) unterstützt. Zur Einhaltung des interdisziplinär festgelegten Hämoglobinziels von 8,5 g/dl wurden der Patientin kumulativ 5 Erythrozytenkonzentrate transfundiert. Die Patientin wurde mit niedermolekularem Heparin therapeutisch antikoaguliert; die Dosierung wurde durch die regelmäßige Bestimmung der Anti-Xa-Aktivität gesteuert (Ziel 0,4–0,6 U/ml). Unter laufender ECMO-Therapie wurde die Antikoagulation mit unfraktioniertem Heparin weitergeführt (Ziel-aPTT 45–50 s). Nach ECMO-Therapie und Normalisierung der COVID-19-typischen Infektionsparameter (CRP 9,2 mg/dl (0-0–5 mg/dl), IL‑6 31,4 pg/ml (0–7 pg/ml), Ferritin 328 µg/l (15–150 µg/l)) wurde niedermolekulares Heparin in prophylaktischer Dosis verabreicht. Die Eigendiurese der Patientin wurde zur Bilanzierung intermittierend mit Furosemid unterstützt.

Während der invasiven Beatmung wurde die Patientin neben Sufentanil zunächst mit Propofol und nach 4 Tagen mit Midazolam und Sevofluran analgosediert. Nach der Extubation entwickelte sie ein produktives Delir, das mit Propranolol, Benperidol und Lormetazepam erfolgreich behandelt wurde. Im weiteren Verlauf äußerte die Patientin wiederholt Angst und beschrieb Panikattacken, in denen sie auch hyperventilierte, sodass wir bis zur Verlegung Lorazepam verabreichten und in Phasen der Agitation zusätzlich Clonidin und Morphin ergänzten. Aufgrund einer posttraumatischen Belastungsstörung (PTBS) wurde eine psychotherapeutische Behandlung begonnen.

Im Anschluss an die intensivmedizinische Therapie wurde die Patientin auf der geburtshilflichen Bettenstation für 13 Tage physiotherapeutisch und psychiatrisch mitbetreut und voll mobilisiert. Mit deutlich gebesserten Laborwerten (Infektwerte CRP 1,48 mg/dl (0–0,5 mg/dl), Leukozyten 8,4/nl (5–10/nl)) und mit intakter Schwangerschaft (32 + 2 SSW) konnte sie 54 Tage nach Symptombeginn in die ambulante frauenärztliche Betreuung entlassen werden. Mit 40 + 6 SSW wurde die Patientin wegen maternaler Erschöpfung und Dyspnoe per sekundärer Sectio von einem eutrophen männlichen Neugeborenen entbunden (3330 g, Apgar 9/10/10). Auf ausdrücklichen Wunsch der Patientin erfolgte der Eingriff in Intubationsnarkose. Vorausgegangen waren 2 Versuche, die Geburt mittels CRB („cervical ripening balloon“) und Prostaglandinpräparaten einzuleiten. Die Untersuchungen des Neugeborenen ergaben keinerlei pathologischen Befund, insbesondere ergab sich in der kraniellen Sonographie kein Hinweis auf eine periventrikuläre Leukomalazie.

## Diskussion

In der Schwangerschaft besteht aufgrund physiologischer Veränderungen eine herabgesetzte antivirale Immunität, sodass eine akute virale Infektion bei der Schwangeren mit einer erhöhten Morbidität und Mortalität einhergeht [2]. Schwangere weisen bei einer SARS-CoV-2-Infektion im Vergleich zu nichtschwangeren Frauen ein höheres Risiko für schwere ante- und postpartale Verläufe auf [3]. Seit Dezember 2020 sind in Deutschland Impfstoffe gegen SARS-CoV‑2 zugelassen, die gut gegen schwere COVID-19-Verläufe schützen, für Schwangere wurde aber zunächst keine Impfempfehlung ausgesprochen. Die Ausbreitung der ansteckenderen Delta-Variante (B.1.617.2) führte jedoch im Vergleich zu den zuvor zirkulierenden Virusvarianten zu einer stärkeren Gefährdung ungeimpfter Schwangerer [4].

Eine SARS-CoV-2-Infektion muss in Bezug auf das ungeborene Kind nicht zwingend zu einer frühzeitigen Entbindung führen [5], sofern eine ausreichende fetale Überwachung gewährleistet ist. Deshalb führten wir tägliche CTG und Sonographien durch. Aus dem interdisziplinären Expertenaustausch mit Kolleginnen und Kollegen aus dem CRONOS-Register [6] wurde berichtet, dass Therapieentscheidungen bei schwangeren Intensivpatientinnen mitunter in kurzer Zeit und unter hohem emotionalem Druck getroffen werden müssen. Darum war es dem interdisziplinären und interprofessionellen Behandlungsteam wichtig, vorab gemeinsame Behandlungsziele zu definieren und für kritische Situationen ein rationales Vorgehen zu konsentieren.

Als „Best-case“-Szenario strebten wir die Entlassung der schwangeren Mutter nach vollständiger Genesung an. Neben dem Überleben von Mutter und Kind stand der Erhalt einer intakten Schwangerschaft bis mindestens in die 32. Schwangerschaftswoche im Vordergrund [7]. Für den Fall eines progredienten Lungenversagens wurden Eskalationsstufen der ARDS-Therapie festgelegt und „Abbruchkriterien“ definiert, die zur vorzeitigen, notfallmäßigen Entbindung führen. Hierzu zählten jeder Anhalt für eine fetale Gefährdung, z. B. vaginale Blutungen oder ein auffälliges CTG. Bei unserer Patientin traten keine COVID-19- oder ECMO-typischen Komplikationen wie pulmonale oder intrazerebrale Blutungen und Thromboembolien, kardiales Pumpversagen oder systemische Infektion auf. Schließlich gab es keinen Anhalt für geburtshilfliche Komplikationen wie Präeklampsie oder Plazentainsuffizienz.

Als Minimalwerte in Bezug auf den mütterlichen Sauerstoffgehalt wurden ein arterieller Sauerstoffpartialdruck (p_a_O_2_) von 70 mm Hg und ein Hämoglobinwert von 8,5 g/dl definiert [8]. Bei Unterschreiten des p_a_O_2_-Minimums trotz nasaler Sauerstoffinsufflation sahen wir zunächst eine High-Flow-Therapie und schließlich eine nichtinvasive Maskenbeatmung vor. Bei anhaltender Hypoxämie zogen wir die Anlage einer venovenösen Wach-ECMO der Intubation vor, um durch Erhalt der Spontanatmung die Ausbildung von zwerchfellnahen Atelektasen zu vermeiden sowie den Einsatz von kreislauf- und fetal-atemdepressiven Analgosedativa zu limitieren. Aufgrund von fachlicher Expertise vor Ort, innerklinischen Ressourcen und hohen Sicherheitsvorkehrungen war in diesem Fall die frühzeitige Anlage einer Wach-ECMO-Therapie umsetzbar. In ähnlichen Fällen wäre für die meisten Schwangeren vermutlich zunächst die konventionelle Beatmungstherapie das geeignetere Vorgehen, um ein adäquates Sauerstoffangebot bereitzustellen. Allerdings zeigen Fallserien und Multizenterstudien, dass die Komplikationsrate für Mutter und Kind mit zunehmender Invasivität der Beatmung zunimmt [3, 12].

Der Einsatz einer ECMO-Therapie in der Schwangerschaft muss nach wie vor als experimentelles Verfahren mit mitunter schlechtem fetalem Outcome angesehen werden [9]. Die Verlegung in ein Zentrum mit geburtshilflicher und neonatologischer Kompetenz hat dazu geführt, dass eine interdisziplinäre Behandlung möglich war. Die Intubation oder der Beginn einer ECMO-Therapie muss eine Notfallentbindung ebenso wenig triggern, wie eine 135°-Lagerung im wachen oder im intubierten Zustand [10]. Insbesondere die Bauchlagerung in der Schwangerschaft wurde in einem Review von Cavalcante et al. als „sicher, zuverlässig und komfortabel“ beschrieben, wenn es um das klinische Management von schwangeren Frauen mit ARDS- und COVID-assoziierten Infektionen geht [11].

Die COVID-19-ARDS-Therapie folgte den nationalen Therapieempfehlungen und -leitlinien. Davon abweichend wurde zur Kortikoidtherapie nicht Dexamethason, sondern das weniger plazentagängige Prednisolon verabreicht und die Antikoagulation in der extern begonnenen therapeutischen Dosis weitergeführt. Die Lungenreifeinduktion mit Betamethason wurde deshalb zusätzlich verabreicht. In der aktuellen AWMF-S2k-Leitlinie „SARS-CoV‑2 in der Schwangerschaft, Geburt und Wochenbett“ wird empfohlen, SARS-CoV-2-infizierten Schwangeren die identischen Indikationen zur Gabe von Kortikosteroiden analog zu Nicht-Schwangeren zur Anwendung kommen zu lassen [5].

Während des stationären Aufenthalts sahen wir keinen Trigger für eine dringliche Beendigung der Schwangerschaft. Nach Entlassung der Mutter wurde die Schwangerschaft unter engmaschigen ambulanten Kontrollen fortgeführt und nach Terminüberschreitung und Einleitung der Geburt ein gesundes Kind per sekundärer Sectio entbunden.

## Fazit für die Praxis

- Die venovenöse ECMO(Extrakorporale Membranoxygenierung)-Therapie ist bei schwangeren Patientinnen mit schwerem Verlauf eines COVID-19-ARDS(Acute Respiratory Distress Syndrome) eine Therapieoption, die nicht zwingend mit der Beendigung der Schwangerschaft einhergehen muss.
- Essenziell ist ein interdisziplinär und interprofessionell konsentiertes Überwachungs- und Therapiekonzept, das auch ein Eskalationsschema für das Unterschreiten von Behandlungszielen umfasst.
- Die Empfehlungen für die Therapie eines COVID-19-ARDS können mit individuellen Anpassungen auch bei Schwangeren angewendet werden.
